# 35.1 ONLY A SMALL PROPORTION OF PATIENTS WITH FIRST EPISODE PSYCHOSIS COME VIA PRODROMAL SERVICES: A RETROSPECTIVE SURVEY OF A LARGE UK MENTAL HEALTH PROGRAMME

**DOI:** 10.1093/schbul/sby014.147

**Published:** 2018-04-01

**Authors:** Olesya Ajnakina, Craig Morgan, Charlotte Gayer-Anderson, Sherifat Oduola, Francois Bourque, Robin Murray, Anthony David

**Affiliations:** Institute of Psychiatry, Psychology & Neuroscience, King’s College London

## Abstract

**Background:**

Little is known about patients with a first episode of psychosis (FEP) who had first presented to prodromal services with an “at risk mental state” (ARMS) before making the transition to psychosis. We set out to identify the proportion of patients with a FEP who had first presented to prodromal services in the ARMS state, and to compare these FEP patients with FEP patients who did not have prior contact with prodromal services.

**Methods:**

In this study information on 338 patients aged ≤37 years who presented to mental health services between 2010 and 2012 with a FEP was examined. The data on pathways to care, clinical and socio-demographic characteristics were extracted from the Biomedical Research Council Case Register for the South London and Maudsley NHS Trust.

**Results:**

Over 2 years, 14 (4.1% of n=338) young adults presented with FEP and had been seen previously by the prodromal services. These ARMS patients were more likely to enter their pathway to psychiatric care via referral from General Practice, be born in the UK and to have had an insidious mode of illness onset than FEP patients without prior contact with the prodromal services.

**Discussion:**

In the current pathways to care configuration, prodromal services are likely to prevent only a few at-risk individuals from transitioning to psychosis even if effective preventative treatments become available.

